# Dibenzazepine Attenuates Against Cisplatin-Induced Nephrotoxicity in Rats: Involvement of NOTCH Pathway

**DOI:** 10.3389/fphar.2020.567852

**Published:** 2020-12-14

**Authors:** Rana H. Abd El-Rhman, Reem N. El-Naga, Amany M. Gad, Mariane G. Tadros, Sherifa K. Hassaneen

**Affiliations:** ^1^ Department of Pharmacology, Egyptian Drug Authority (ED), Giza, Egypt; ^2^ Department of Pharmacology and Toxicology, Faculty of Pharmacy, Ain Shams University, Cairo, Egypt; ^3^ Department of Pharmacology and Toxicology, Faculty of Pharmacy, Sinai University, East Kantara Branch, New City, El Ismailia, Egypt

**Keywords:** cisplatin, nephrotoxicity, dibenzazepine, notch, inflammation

## Abstract

Cisplatin is one of the standard anti-cancer agents that are used to treat variety of solid tumors. Nevertheless, due to the accumulation of cisplatin in the renal epithelial cells, nephrotoxicity was found to be the main side effect that limits its clinical use. The current study was conducted to assess the potential nephroprotective effect of dibenzazepine, a Notch inhibitor, against cisplatin-induced nephrotoxicity in rats as well as the possible mechanisms underlying this nephroprotection. The rats were pre-treated with 2 mg/kg dibenzazepine for 7 days before giving a single nephrotoxic dose of cisplatin (7 mg/kg). Cisplatin induced acute nephrotoxicity, where blood urea nitrogen and serum creatinine levels were significantly increased. Besides, lipid peroxidation was markedly elevated and the levels of reduced glutathione and catalase were significantly reduced. Also, the tissue levels of the pro-inflammatory mediators; IL-1β, TNF-α, and NF-kB, were significantly increased in the cisplatin group. The pre-treatment with dibenzazepine significantly mitigated the nephrotoxic effects of cisplatin, the oxidative stress and inflammatory status as well as decreased caspase-3 expression, as compared to the cisplatin group. Furthermore, the up-regulation of Notch-1 and Hes-1 was found to be involved in cisplatin-induced nephrotoxicity and their expression was significantly reduced by dibenzazepine. The nephroprotective effect of dibenzazepine was further confirmed by the histopathological assessment. Moreover, dibenzazepine pre-treatment of hela and PC3 cells *in vitro* did not antagonize the cisplatin anti-cancer activity. In conclusion, these findings show that dibenzazepine provides protection against cisplatin-induced nephrotoxicity. Moreover, the up-regulation of the Notch pathway was shown to play a role in the pathogenesis of cisplatin-induced renal injury.

## Introduction

Cisplatin is one of the most noticeable successes in “the war on cancer” (Arany and Safirstein, 2003; Wang and Lippard, 2005). It is an anti-tumor drug which represents one of the standard anti-cancer agents used to cure many solid tumors (Dasari and Tchounwou, 2014). In spite of the positive effects of platinum compounds, patients taking these agents experience severe side effects that restrict the dose (Ruggiero et al., 2013). It was proved that the prominent dose-limiting side-effect of cisplatin is the renal toxicity that happens at doses lower than that could damage other organs (Azu et al., 2010; Shahbazi et al., 2015; Dugbartey et al., 2016). Despite using protective measures, the consecutive cisplatin dosing results in cumulative and irreversible nephrotoxicity (Crona et al., 2017). Remarkably, the oxidative stress is actively involved in the pathogenesis of cisplatin-induced acute renal injury and immensely drives to apoptotic cell death both *in vitro* (Yang et al., 2019) and *in vivo* (Badawy et al., 2019; Soetikno et al., 2019). Also, there are multiple suggestion about the involvement of pro-inflammatory cytokines in the pathogenesis of cisplatin-induced nephrotoxicity (Gao et al., 2019; Güntürk et al., 2019; Iwakura et al., 2019; Michel and Menze, 2019; Soetikno et al., 2019). Indeed, searching for other pathways that may be engaged in the pathogenesis of cisplatin renal injury is required for finding new promising protective strategies against this deleterious effect.

The Notch pathway plays an important role in cell-cell communication (Fortini, 2009; Jolly et al., 2015). Besides, the Notch signaling was found to be deregulated in many types of cancer (Moserle et al., 2010; Wu et al., 2010; Yin et al., 2010; Aster et al., 2017; Meurette and Mehlen, 2018; Kontomanolis et al., 2018). Indeed, this pathway is involved in the proliferation, differentiation, and self-renewal of cancer stem cells which are responsible for the chemo- and radio-resistance (Wang et al., 2008). The Notch pathway gets activated upon ligand-receptor interaction, which is followed by two enzymatic cleavages occur, by the alpha- and the gamma-secretase, respectively (Muller et al., 2007). It was shown that the Notch pathway regulates the expression of multiple target genes, such as Hairy enhancer of split (Hes-1) (Wu et al., 2012). Interestingly, this pathway was found to play an important role in renal ischemia as well as reperfusion injury-associated inflammation and apoptosis (Huang et al., 2011). Also, the expression of the intracellular domain of Notch-1 was shown to be significantly increased in the glomerular epithelial cells in diabetic nephropathy (Niranjan et al., 2008). Moreover, Notch was shown to play a role in streptozocin-induced kidney injury (Jiandong et al., 2009). However, the role of Notch signaling in the pathogenesis of cisplatin-induced nephrotoxicity has not been investigated before.

Dibenzazepine (DBZ) is a gamma-secretase inhibitor that interferes with the Notch signaling pathway and effectively prevent the activation of all Notch receptors by inhibiting this final enzymatic cleavage (Nowell and Radtke, 2017). Particularly, the gamma-secretase inhibitors have been shown to have both anti-inflammatory and anti-proliferative properties (Kang et al., 2009; Piggott et al., 2011; Hans et al., 2012; Pan et al., 2012; Zhao et al., 2019; Michelon et al., 2020). Notably, DBZ was found to have anti-cancer activity in a variety of cancer cells (Nickoloff et al., 2003; Curry et al., 2005; Van et al., 2005; Katoh, 2007; Shih and Wang, 2007; Al-Qawasmeh et al., 2009). Moreover, Xiao et al. (2014) had found that DBZ attenuated the kidney fibrosis induced by the unilateral ureter obstruction in mice. Accordingly, DBZ might be a promising agent to ameliorate cisplatin-induced renal injury.

Therefore, the aim of the current research was to investigate, for the first time, the potential nephroprotective effect of DBZ against cisplatin-induced acute nephrotoxicity in rats. Also, the probable mechanisms underlying this effect were explored; particularly its effects on oxidative stress, inflammation, apoptosis, and the Notch pathway signaling.

## Material and Methods

### Material

Cisplatin was purchased from Merk Ltd., Cairo, Egypt and supplied as a clear liquid (1 mg/ml). Dibenzazepine was purchased from Sigma Chemical Co. (St. Louis, MO, United States). Cisplatin was injected intraperitoneally as a single dose of 7 mg/kg according to (El-Naga, 2014; Parhizgar et al., 2016). Dibenzazepine was administered for 12 days. The dose was selected as previously reported (Zheng et al., 2013) as well as from the pilot experimental trials of the present study. All chemicals and solvents were of the highest grade commercially available.

### The Animals

Male Sprague-Dawley albino rats (150–200 g) were obtained from the breeding colony and then maintained at the animal house of the National Organization for Drug Control and Research (NODCAR, Giza, Egypt). Animals had free access to food and water. They were maintained at 21–24°C and 40–60% relative humidity with 12-h light–dark cycle. Animals were subjected to 1 week adaptation period in the animal house before the beginning of the experiments. Experimental procedures were conducted in accordance with the international ethical guidelines for investigations in laboratory animals and were approved by the Research Ethical Committee of Faculty of Pharmacy, Ain shams University, Cairo, Egypt (serial number: Master No. 86).

### The Cell Lines

PC3 and Hela human cancer cell lines were obtained frozen in liquid nitrogen from American Type Culture Collection (ATCC). The cell line was maintained in Faculty of Pharmacy, Al-Azhar University, Cairo, Egypt by serial sub-culturing. Cells were grown as “monolayer culture” in RPMI-1640 medium supplemented with 10% (v/v) fetal bovine serum and 100 U/ml penicillin and 100 μg/ml streptomycin antibiotic. The cell lines were incubated at 37°C in 5% CO_2_–95% air.

### The *In Vivo* Part

#### The Experimental Design

Animals were divided randomly into four groups (ten animals per group) and treated for 12 days as follows; the first group served as control where rats received DMSO/corn oil mixture (1:9) i.p., which was used as a vehicle for DBZ. The second group was given the vehicle i.p., once daily for 12 days starting 7 days before giving cisplatin (7 mg/kg i.p.) as a single dose to induce nephrotoxicity. The third group was given DBZ at a dose of (2 mg/kg; i.p) once daily for seven consecutive days followed by a single i.p., injection of cisplatin (7 mg/kg) on the 8th day then DBZ administration was continued till the 12th day. The fourth group was given DBZ (2 mg/kg) i.p. once daily for 12 consecutive days. At the end of the experiment, animals were sacrificed then blood samples were collected and serum was separated by centrifugation for 20 min at 4,000 rpm using high speed centrifuge (MPw-350, Warsaw, Poland), and used for measuring blood urea nitrogen (BUN) and serum creatinine. Kidney tissues were dissected out and washed with ice-cold saline. Then, the tissues were homogenized and stored at −80°C till the estimations of the oxidative stress, inflammatory, apoptotic markers as well as the Notch signaling pathway. Additionally, the rest of each kidney was fixed in 10% formol saline for the routine histopathological examination.

#### The Assessment of Nephrotoxicity Markers

For all the experimental groups, the mortality rate and body weights were recorded. In addition, the relative kidney weight was calculated according to the formula: (kidney weight/total body weight) × 100. Colorimetric assay kits (Biodiagnostics, Cairo, Egypt) for the measurement of BUN and serum creatinine levels were used in this study. All procedures were performed according to the manufacturer’s instructions.

#### The Assessment of Oxidative Stress Markers

In the kidney homogenates of the different treatment groups, GSH, MDA levels and catalase activity were assessed. The GSH was assessed according to the method described by (Ellman, 1959). Also, lipid peroxidation was determined by estimating the level of thiobarbituric acid reactive substances (TBARS) measured as MDA, according to the method of (Satoh, 1978). Catalase activity was assessed using catalase assay kit (Biodiagnostic, Cairo, Egypt) in accordance with manufacturer’s instructions.

#### The Assessment of Protein Content

The protein content in the kidney homogenates was determined according to the method of (Gornall et al., 1949).

#### The Assessment of Inflammatory Markers

The involvement of inflammation in cisplatin-induced nephrotoxicity was assessed by measuring IL-1β, TNF-α, and NF-KB tissue levels. The levels of (TNF-α and IL-1β) in kidney homogenate of all groups were measured by using RayBio®Rat TNF-α and RayBio®Rat IL-1β ELISA Kits (RayBiotech, Inc., United States), respectively. The manufacturer’s instructions were precisely followed. The intensity of the color measured at 450 nm using a microplate reader is in proportion to the amount of rat antigen bound in the initial steps. The samples concentrations are then read off the standard curve.

Also, the kidney blocks were used for immunohistochemical assessment of NF-kB. The slides were then blocked with 5% bovine serum albumin in tris buffered saline for 2 h. The section was then immunostained with the primary antibody (rabbit polyclonal IgG to rat NF-kB p65) at a concentration of 1 μg/ml containing 5% bovine serum albumin in tris buffered saline and incubated overnight at 4 °C. After washing the slides with tris buffered saline, the section was incubated with goat anti-rabbit secondary antibody. Section was then washed with tris buffered saline and incubated for 5–10 min in a solution of 0.02% diaminobenzidine containing 0.01% H_2_O_2_. Counter staining was performed using hematoxylin, and the slide was visualized under a light microscope (Buchwalow and Böcker, 2002). Immunohistochemical quantification of positive areas was performed using ImageJ analysis software (ImageJ, 1.50i, NIH, United States).

#### The Assessment of the Apoptotic Marker, Caspase-3

capase-3 level activity was detected in the kidney homogenates using ELISA kit (Cusabio Life Science, Inc., China). The manufacturer’s instructions were followed precisely and the developed color was measured spectrophotometrically at 450 nm immediately.

#### The Assessment of Notch signaling Pathway

Notch-1 and Hes-1 levels in kidney tissue were detected using quantitative reverse transcriptase polymerase chain reaction (qRT-PCR). The procedure was carried out according to the manufacturer’s instructions (Qiagen, United States).

#### The Histopathological Examination

For light microscopy, autopsy samples were taken from the kidney of rats in the different groups and fixed in 10% formol saline for 24 h. Washing was done in tap water then serial dilutions of alcohol (methyl, ethyl and absolute ethyl) were used for dehydration. Specimens were cleared in xylene and embedded in paraffin at 56° in hot air oven for 24 h. Paraffin bees wax tissue blocks were prepared for sectioning at four microns thickness by sledge microtome. The obtained tissue sections were collected on glass slides, deparaffinized and stained by hematoxylin & eosin stain for examination by the light microscope (Banchroft et al., 1996).

### The *In Vitro* Part

#### The Cytotoxicity Assay

The cytotoxicity was determined using 3-(4,5-Dimethylthiazol-2-yl)-2,5-Diphenyltetrazolium Bromide (MTT) Assay (Germain et al., 2010) in order to assess the modulatory effect of DBZ on cisplatin cytotoxic activity. In a 96-well flat-bottomed plate, 5,000 cells/150 μl of cell suspension were used to seed each well. After treatment with various concentrations of drugs, MTT was added to each well and incubated at 37°C. The resulting violet formazan precipitate was solubilized and the absorbance was read at 570 nm using a plate reader. Concentration-response curves were generated and IC50 for each curve was calculated (GraphPad Prism software, version 5).

#### The Statistical Analysis

Data are presented as mean ± SD. Paired *t*-test was used to compare the change in weight in the same group before and after receiving the treatment. Multiple comparisons were performed using one-way ANOVA followed by either Dunnett or Tukey–Kramer test for post hoc analysis, as appropriate. The 0.05 level of probability was used as the criterion for significance. All statistical analyses were performed using the SPSS version 16 (Chicago, IL, United States), while the graphs were drawn using a prism computer program (GraphPad software Inc., V5, San Diego, CA, United States).

## Results

### The *In Vivo* Part

***The nephrotoxicity markers*** *.* No deaths were observed in the control and dibenzazepine-only treated groups, while the mortality rate was significantly increased to 40% in the cisplatin-injected group. On the other hand, pre-treatment of cisplatin-injected rats with dibenzazepine significantly reduced the mortality rate to 20% only. Indeed, the group injected with cisplatin showed a significant decrease in the body weight of rats by 17.3%, as compared to the original body weight. However, no significant changes in body weights were observed in all other groups. There was a significant increase in relative kidney weight by 73.2% in the cisplatin-treated rats, as compared to the control group. Notably, DBZ pre-treatment greatly ameliorated cisplatin-induced changes in body weight and relative kidney weight (Table 1).

**TABLE 1:** The effects of pre-treatment with dibenzazepine on the mortality rate, body weight, relative kidney weight, serum creatinine, and blood urea nitrogen in the cisplatin-injected rats.

Treated groups	No of dead rats	Body weight (g)		Relative kidney weight	Blood urea nitrogen (mg/dl)	Serum creatinine (mg/dl)
		Before treatment	After treatment			
Control	0/10^a^	192.00 ± 11.61	213.20 ± 24.09^b^	0.56 ± 0.05^a^	12.81 ± 0.65^a^	0.65 ± 0.03^a^
Cisplatin	4/10^c^	216.00 ± 10.81	178.70 ± 6.65^b^	0.97 ± 0.06^c^	85.01 ± 1.27^c^	4.88 ± 0.45^c^
Cisplatin/Dibenzazepine	2/10^a,c^	202.00 ± 3.50	227.20 ± 19.96^b^	0.72 ± 0.09^a,c^	36.65 ± 2.60^a,c^	1.63 ± 0.16^a,c^
Dibenzazepine	0/10^a^	256.20 ± 18.76	253.20 ± 33.91	0.60 ± 0.02^a^	15.60 ± 0.77^a^	0.79 ± 0.06^a^

Each value indicates the mean ± SD of six observations.

a Significantly different from the control or cisplatin group, respectively at *p* < 0.05 using ANOVA followed by Tukey–Kramer, as a post-hoc test.

b Statistically significant when compared to the values obtained before treatment, *p* < 0.05 using paired *t*-test.

c Significantly different from the control or cisplatin group, respectively at *p* < 0.05 using ANOVA followed by Tukey–Kramer, as a post-hoc test.

Moreover, rats injected with cisplatin showed marked elevations in the levels of serum creatinine and blood urea nitrogen 650.8 and 563.6%, respectively, when compared to the control rats. Notably, these levels were significantly improved in the DBZ pre-treated group where serum creatinine and blood urea nitrogen were significantly decreased by 66.6 and 56.8%, respectively, when compared to the group that received cisplatin only.

***The oxidative stress markers*** *.* The effect of the different treatment groups on GSH, MDA and catalase levels are shown in Table 2. Reduced glutathione and catalase levels were significantly reduced in cisplatin-injected rats by 50.3 and 58.1%, respectively, while MDA level was markedly increased by 74.2%, as compared to control values. Notably, GSH and catalase levels in rats pre-treated with DBZ showed a significant increase reaching 70.4 and 95.5%, respectively, while MDA level was decreased by 30.6%, as compared to the cisplatin group. Moreover, GSH, catalase and MDA remains unchanged in rats treated with DBZ only.

**TABLE 2:** The effects of pre-treatment with dibenzazepine on the oxidative stress markers in the cisplatin-injected rats.

Treated groups	GSH (mg/mg protein)	MDA (nmol/mg protein)	CAT (mg/mg protein)
Control	98.31 ± 4.68^a^	39.89 ± 1.35^a^	2.10 ± 0.23^a^
Cisplatin	48.90 ± 5.03^b^	69.50 ± 4.06^b^	0.88 ± 0.07^b^
Cisplatin/Dibenzazepine	83.35 ± 1.09^a,b^	48.25 ± 1.66^a,b^	1.72 ± 0.06^a,b^
Dibenzazepine	94.44 ± 3.23^a^	42.32 ± 2.48^a^	1.93 ± 0.04^a^

Each value indicates the mean ± SD of six observations.

a Significantly different from the control or cisplatin group, respectively at *p* < 0.05 using ANOVA followed by Tukey–Kramer, as a post-hoc test.

b Significantly different from the control or cisplatin group, respectively at *p* < 0.05 using ANOVA followed by Tukey–Kramer, as a post-hoc test.

GSH, glutathione; MDA, malondialdehyde; CAT, catalase.

**FIGURE 1:**
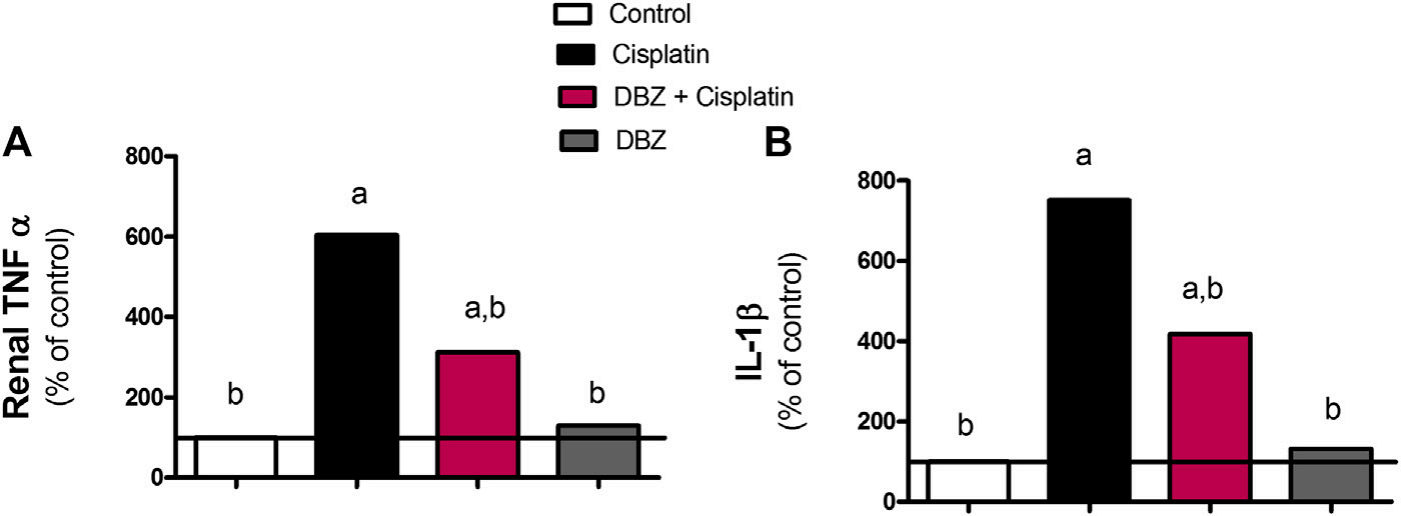
The effect of dibenzazepine on the expression of **(A)** tumor necrosis factor and **(B)** interleukin -1 beta in renal tissues in cisplatin-injected rats. Data presented as % of control value (*n* = 6 rats, per group). **a** or **b**: Statistically significant from the control or the cisplatin group, respectively, *p* < 0.05 using ANOVA followed by Tukey-Kramer as post-hoc test.

***The inflammatory markers***. The levels of the pro-inflammatory markers, TNF-α and IL-1β, in renal tissues were assessed in the different treatment groups (Figure 1). The administration of cisplatin markedly increased tissue levels of TNF-α and IL-1β by 504.6 and 651.4%, respectively, as compared to the control values. This significant increase in the assessed pro-inflammatory markers was reduced in the DBZ -pre-treated group by 48.5 and 44.4%, respectively, as compared to the cisplatin-treated rats. Besides, animals receiving DBZ only showed no significant changes in TNF-α and IL-1β, as compared to the control group.

**FIGURE 2:**
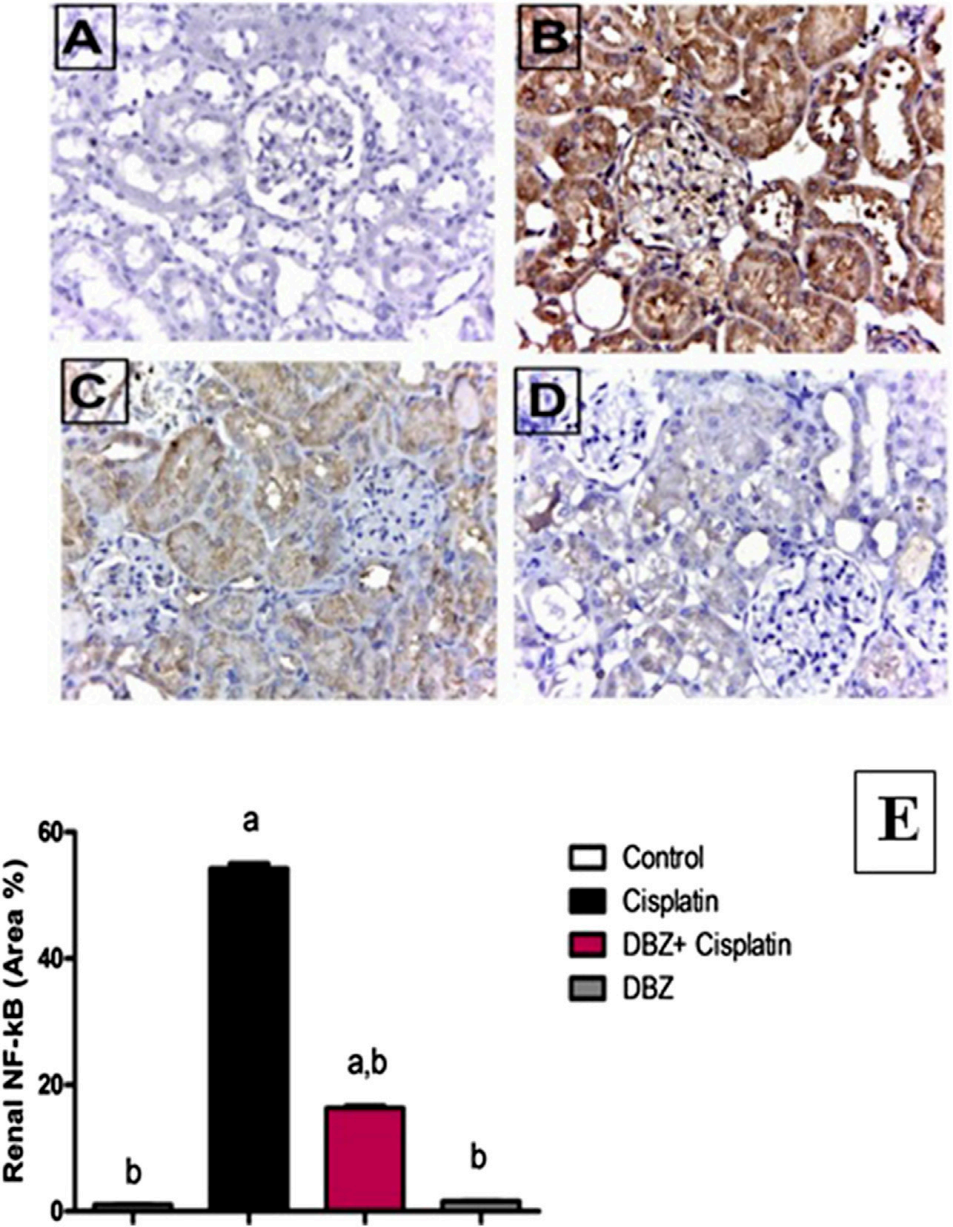
The effect of dibenzazepine (2 mg/kg/day given i.p.) on NF-kB positive cells expression of kidney {X 400}. **(A)** Photomicrograph of kidney section of control group showing no expression of NF-kB. **(B)** Photomicrograph of kidney section of cisplatin group showing high immune-reactivity in the glomerular tuft and proximal tubules. **(C)** Photomicrograph of kidney section of pre-treated with DBZ group showing very low immune-reactivity in the glomerular tuft and proximal tubules. **(D)** Photomicrograph of kidney section of DBZ only showing no expression of NF-kB. **(E)** Quantitative image analysis for immunohistochemical staining expressed as area percent across 10 different fields for each rat section. Values are given as mean ± SD of six observations, **a** or **b**: Statistically significant from the control or the cisplatin group, respectively at *p* < 0.05 using ANOVA followed by Tukey–Kramer as a post-hoc test. DBZ, dibenzazepine. *Scale bar for all the previous photomicrographs is 25 μm.

The immunohistochemical analysis of NF-kB expression in the renal tissues revealed that the control rats showed minimal expression of NF-kB (Figures 2A,E). While, cisplatin markedly induced NF-kB expression, where the area of immune-reactivity reached 54% (Figures 2B,E). On the other hand, the DBZ pre-treated group showed a significant reduction in NF-kB expression by 70.4%, as compared to the cisplatin group (Figures 2C,E). Furthermore, the animals that received DBZ only showed no significant change in NF-kB expression, as compared to the control group (Figures 2D,E).

**FIGURE 3:**
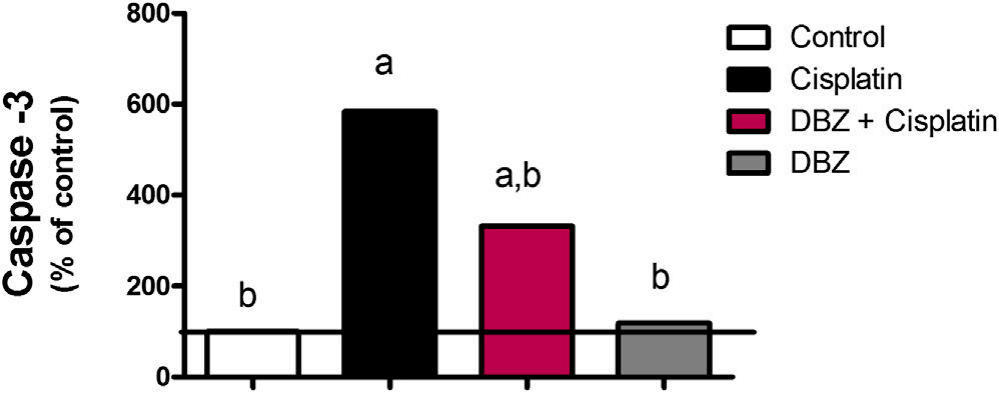
The effect of dibenzazepine on caspase-3 levels in renal tissues in cisplatin-injected rats. Data presented as % of control value (*n* = 6 rats, per group). **a** or **b**: Statistically significant from the control or the cisplatin group, respectively, *p* < 0.05 using ANOVA followed by Tukey-Kramer as post-hoc test.

***Caspase-3***. As shown in Figure 3, cisplatin-treated group showed a significant increase in caspase-3 level by 483.7%, as compared to that of the control rats. On the other hand, pre-treatment of cisplatin-injected rats with DBZ significantly reduced caspase-3 levels by 43.2%, as compared to the cisplatin group.

**FIGURE 4:**
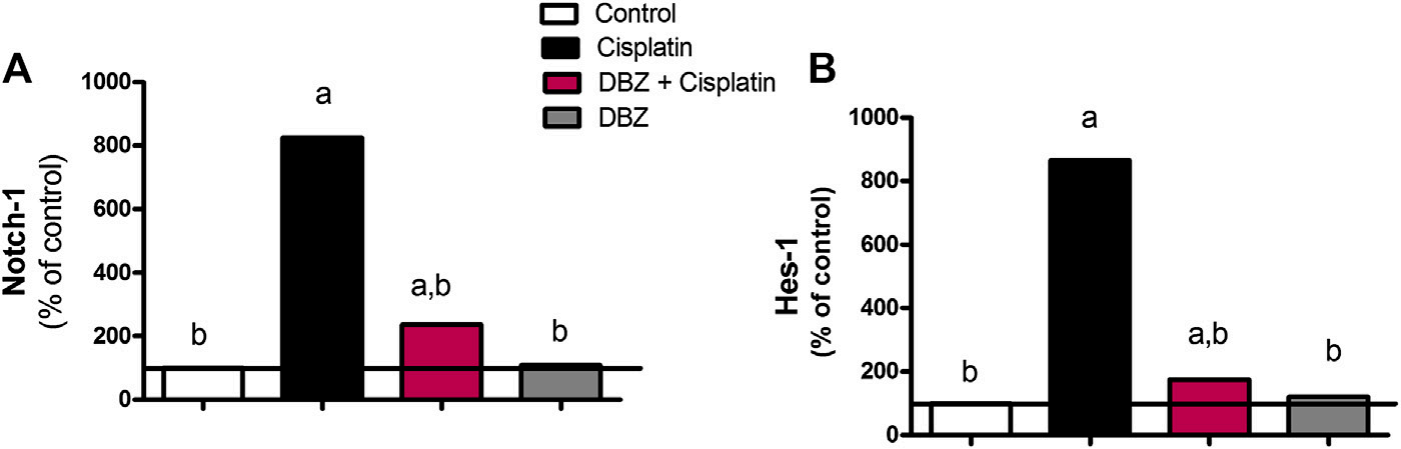
The effect of dibenzazepine on the mRNA levels of **(A)** Notch-1 and **(B)** Hes-1 in renal tissues in cisplatin-injected rats. Data presented as % of control value (*n* = 6 rats, per group). **a** or **b**: Statistically significant from the control or the cisplatin group, respectively, *p* < 0.05 using ANOVA followed by Tukey-Kramer as post-hoc test.

***The Notch signaling pathway***. Cisplatin-injected rats showed a marked increase in the mRNA levels of Notch-1 and Hes-1 level by 725 and 765%, respectively, as compared to the control values. However, the Notch-1 and Hes-1 mRNA levels were significantly reduced by DBZ pre-treatment by 71.4 and 79.8%, respectively, as compared to the cisplatin treated rats. Compared to the control values, animals that received DBZ only did not show any significant change in the mRNA levels of Notch-1 and Hes-1 (Figure 4).

**FIGURE 5:**
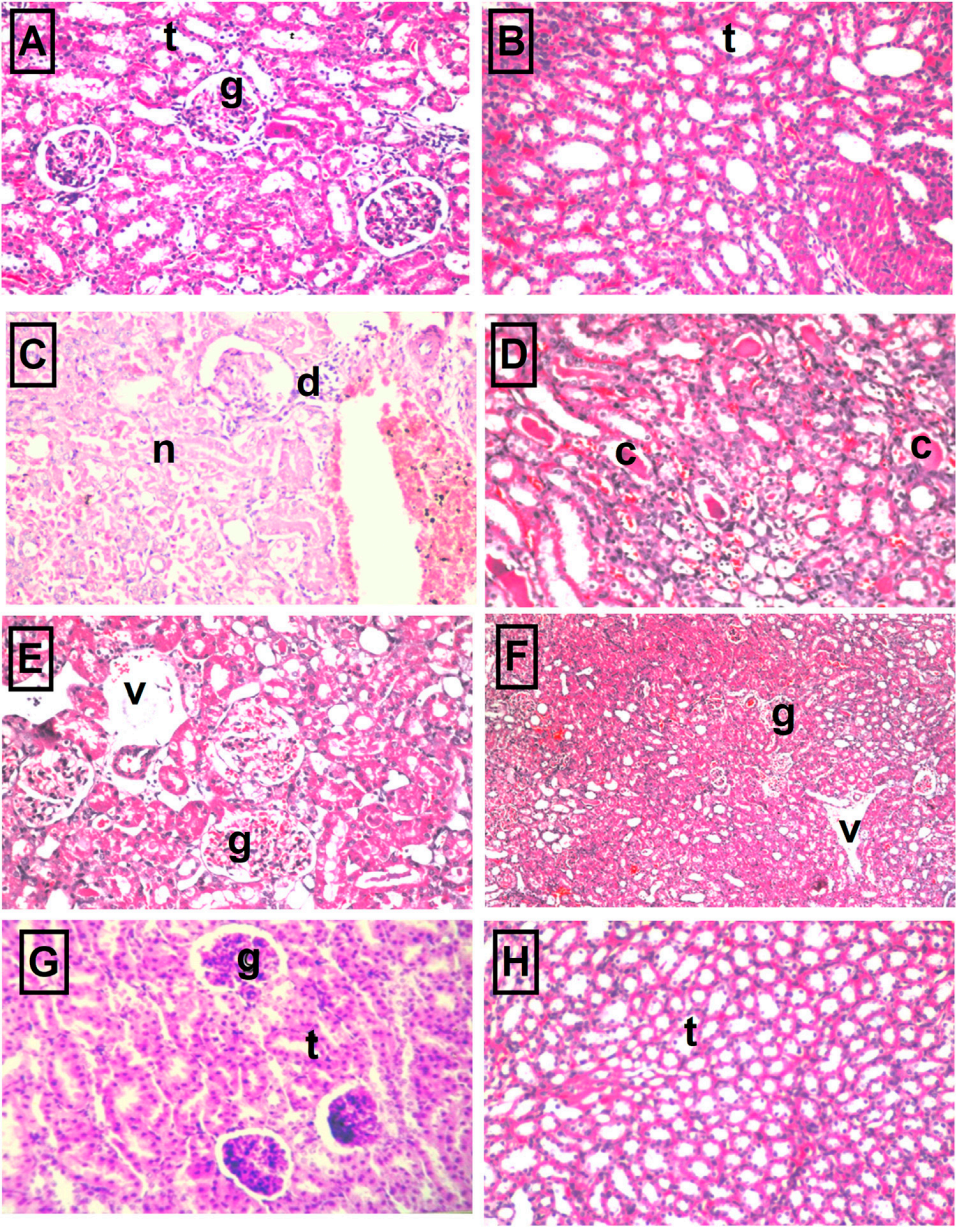
The representative photomicrographs of kidney sections stained with hematoxylin–eosin stain: **(A,B)** Sections taken from kidneys of the control group showing normal glomeruli (g) and tubules (t) at the cortex portion and normal histological structure of the tubules (t) at the corticomedullary portion as well. **(C,D)** Sections taken from kidneys of the cisplatin group showing necrosis of epithelial lining renal tubules and presence of renal cast in the lumen of renal tubules as well as atrophy of glomerular tuft and distension of Bowman’s space (arrow). **(E,F)** Sections were taken from kidney specimens of rats treated with dibenzazepine and cisplatin showing no degeneration or necrosis where only mild congestion in blood vessels (v) and glomerular tufts (g) were observed. **(G,H)** Sections taken from kidney specimens of rats treated with dibenzazepine only showing normal histological structure of the glomeruli (g) and tubules (t) at the cortex.

***The kidney pathology*** *.* Table 3 and Figure 5 show the different histopathological alterations observed in the kidney specimens taken from the different treatment groups. The histopathological examination of kidney sections of the control group showed normal histological structure (Figures 5A,B), while in the cisplatin-injected rats, degenerative changes and coagulative necrosis were noticed in the lining epithelium of the tubules at the cortex and medulla with the formation of eosinophilic casts in the tubular lumen (Figures 5C,D). In contrast, no degeneration or necrosis was observed in the DBZ pre-treated group (Figures 5E,F). While the administration of DBZ only showed no histopathological alteration in the glomeruli and tubules at the cortex and the tubules in both corticomedullary and medullary portions (Figures 5G,H).

**TABLE 3:** The severity of histopathological alterations in the kidney specimens taken from the different experimental groups.

Histopathological alteration	Control	Cisplatin	Cisplatin/Dibenzazepine	Dibenzazepine
Degenerative change (R.T)	−	+++	−	−
Coagulative necrosis (R.T)	−	+++	−	−
Renal casts	−	++	−	−
Congestion in the glomerular tufts	−	++	+	−
Congestion in blood vessels	−	+	+	−

+++ Severe ++ Moderate + Mild **-** Nil.

### The *In Vitro* Part

#### The Effect of Pre-Treatment With Dibenzazepine on Cisplatin Cytotoxic Activity

**FIGURE 6:**
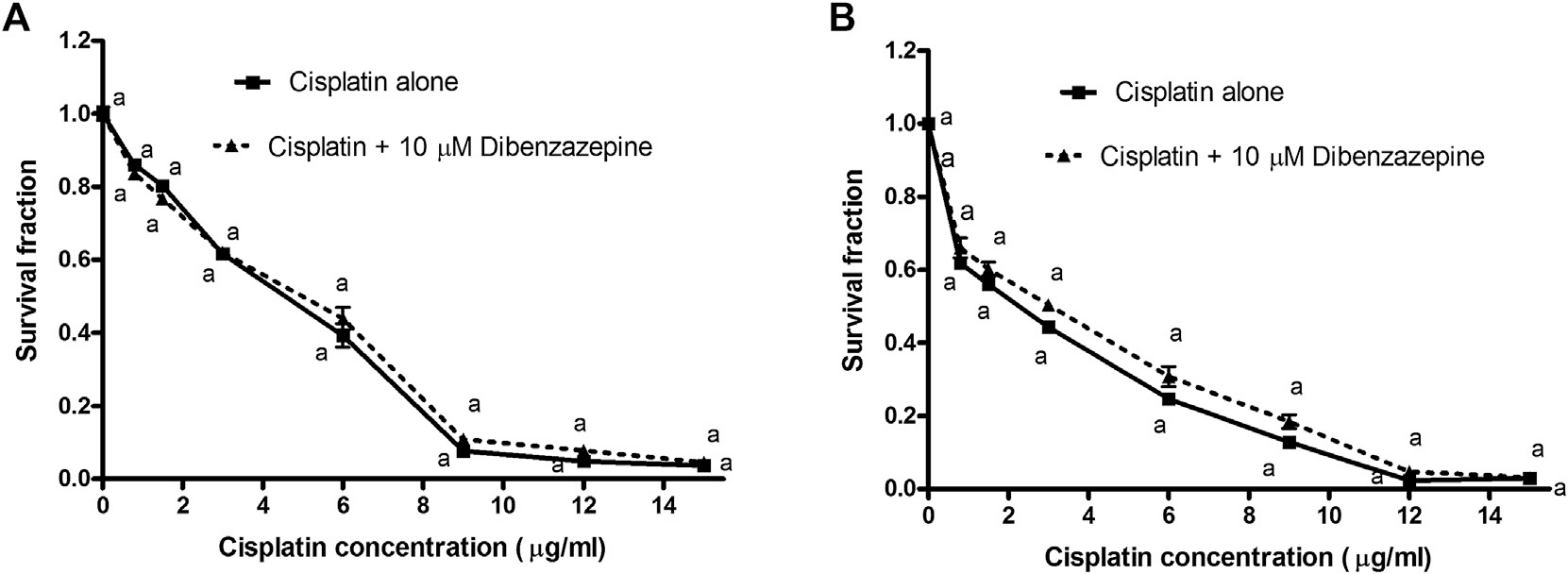
**A)** Cytotoxicity of various concentrations of cisplatin alone or in combination with 10 μM dibenzazepine in PC3 cancer cell line. ^a^ *p* < 0.05: Statistically significant when compared to the control value using ANOVA followed by Dunnett test as post-hoc test. **(B)** Cytotoxicity of various concentrations of cisplatin alone or in combination with 10 μM dibenzazepine in Hela cancer cell line. ^a^ *p* < 0.05: Statistically significant when compared to the control value using ANOVA followed by Dunnett test as post-hoc test.

The cell viability was expressed as the survival fraction compared with the untreated control cells as shown in Figure 6. The MTT assay revealed that the treatment of PC3 and Hela human cancer cells with different concentrations of cisplatin for 24 h significantly decreased the survival fraction of cells in a concentration-dependent manner. Cisplatin IC50 was obtained from the fitted survival curve and was found to be 4.6 and 2.3 μg/ml for PC3 and Hela human cancer cells, respectively. It was shown that the pre-treatment of PC3 and Hela cancer cells with DBZ for 24 h, before cisplatin addition, did not alter cisplatin cytotoxic activity.

## Discussion

The current study was the first one to investigate the potential protective effect of DBZ against cisplatin-induced acute nephrotoxicity in rats. Also, the probable mechanisms underlying this nephroprotective effect were explored particularly; the effects on the oxidative stress markers, inflammatory mediators, apoptosis as well as Notch signaling pathway. This study showed that the total body weight of rats injected with cisplatin at a dose of 7 mg/kg was significantly reduced, as compared to the rats given the vehicle only. The renal injury that was instigated by cisplatin was manifested by the prominent increase in the mortality rate, relative kidney weight, BUN, and serum creatinine levels, as compared to the control animals, suggesting the occurrence of acute renal failure. The histopathological findings demonstrated that the administration of cisplatin induced a severe degenerative change. Also, coagulative necrosis was noticed in the lining epithelium of the tubules at the cortex and medulla together with the formation of eosinophilic casts in the tubular lumen. In the present study, the results showed that the intraperitoneal administration of DBZ (2 mg/kg) prior to and after cisplatin markedly attenuated cisplatin-induced changes in the body weight, relative kidney weight, mortality rate, nephrotoxicity markers, and the histological architecture of the kidney.

The second step was conducted to study the mechanisms underlying the nephroprotective effects of DBZ, including its effects on the oxidative stress, inflammation, apoptosis as well as the Notch signaling pathway. The previous study conducted by (Demkow et al., 2011) reported that cisplatin induces its acute kidney injury through the induction of pro-oxidant/antioxidant imbalance. This status increases the free radicals production and decreases the antioxidants production, where the free radicals damage the lipid components of the cell membrane via the peroxidation and denaturation of its proteins (Chtourou et al., 2015). The findings of this study denoted that the reduction in the renal GSH levels, the increase in MDA levels besides the reduction of catalase enzymatic activity are involved as signs of injury affirming the oxidative stress triggered by cisplatin treatment. Immensely, these signs are confirming the results previously stated in a number of studies (Omar et al., 2016).

Notably, the pre-treatment of cisplatin-injected group with DBZ ameliorated the oxidative stress by significantly increasing GSH and catalase levels and lowering MDA levels in the kidney homogenate. These findings were in accordance with previous studies showing that agents with anti-oxidant activity, such as selenium, onion oil, hesperidin and rutin, have potential protective effects against cisplatin-induced nephrotoxicity (Pan et al., 2009; Kamel et al., 2014; Kamel et al., 2015; Badawy et al., 2019; Soetikno et al., 2019).

The Notch-1 signaling pathway plays an important role in the regulation of lipid oxidation. So, the inhibition of gamma-secretase or Notch signaling pathway can modulate the expression of fatty acid oxidation genes and may provide therapeutic strategies to prevent and treat fatty liver disease associated with obesity (Song et al., 2016). It was found that in Alzheimer disease, hydrogen peroxide induced the up-regulation of Notch-1 possibly due to the activation of enzymes involved in Notch-1 cleavage and gamma-secretase activation (Shen et al., 2008). Accordingly, it was expected that the pharmacological inhibition of gamma-secretase activity will be worthy (Xu et al., 2009; Borghese et al., 2010).

In addition to the oxidative stress, inflammation was proven to play an essential role in the pathogenesis of cisplatin-induced renal injury. Previous studies showed that the expression of inflammatory cytokines is elevated in the kidney after cisplatin injury (Miller et al., 2010). The levels of several pro-inflammatory cytokines, such as TNF-α and IL-1β, were found to be elevated in the urine of cisplatin-treated mice (Gao et al., 2019; Güntürk et al., 2019; Iwakura et al., 2019; Michel and Menze, 2019; Soetikno et al., 2019). In this study, the cisplatin-treated group showed a marked increase in pro-inflammatory cytokines tissue levels; IL-1β and TNF-α. On the other side, DBZ exerted anti-inflammatory effects where the group that received DBZ pre-treatment showed a significant decrease in the kidney contents of the assessed pro-inflammatory markers. Particularly, gamma-secretase inhibitors have been shown to have both anti-inflammatory and anti-proliferative properties (Kang et al., 2009; Piggott et al., 2011; Hans et al., 2012; Pan et al., 2012; Zhao et al., 2019; Michelon et al., 2020). These include the inhibition of macrophage and T cell infiltration, M1/M2 transition and cytokine expression (Jiandong et al., 2009; Piggott et al., 2011; Hans et al., 2012). Indeed, the treatment with anti-inflammatory agents is a promising strategy in reducing cisplatin-induced renal dysfunction and decreasing the histological evidence of injury (El-Naga, 2014; El-Naga and Mahran, 2016; Gao et al., 2019; Güntürk et al., 2019; Iwakura et al., 2019; Michel and Menze, 2019; Soetikno et al., 2019).

Furthermore, the present investigation shows another explanation for the renoprotection effect conferred by DBZ that is linked to the suppression of NF-κB, which was elevated in the cisplatin-injected group. Indeed, the NF-κB plays an important role in various biological processes, including immune response, inflammation, regulation of cell differentiation, proliferation and survival (Gerondakis et al., 1999; Pasparakis et al., 2006). As a consequence, dysregulation of NF-κB activity is linked to inflammatory disorders, autoimmune and metabolic diseases, as well as cancer (Kumar et al., 2004; Courtois and Gilmore, 2006). In current study, cisplatin-treated group showed a marked elevation in NF-κB immune-reactivity. Nevertheless, this was significantly attenuated in the group that received DBZ pre-treatment.

Upon investigating the cellular pathways of cisplatin injury to kidney, apoptosis was found to be considerably involved. Indeed, the intrinsic mitochondrial pathways as well as death receptors pathways are activated in renal cells by cisplatin injection (Oh et al., 2014; Topcu-Tarladacalisir et al., 2016). The relationship between cisplatin dose and caspase-3 activity induction was evaluated by Lau (1999). This study showed that following the exposure to cisplatin for 12 h, there was a dose-dependent increase in caspase-3 activity induction. However, the induced caspase-3 activities began to decline as the cisplatin concentration was increased beyond 50 mM. These data indicate that cisplatin treatment results in a significant increase in caspase-3 enzymatic activity. Our results showed that cisplatin injection in rats triggered apoptotic cell death that was represented by significantly enhancing the active form of the executive caspase, caspase-3, in renal tissues. Moreover, the effect of cisplatin on the level of capsase-3 enzyme was notably reduced by DBZ pre-treatment.

Finding new pathways that may be involved in the pathogenesis of cisplatin renal injury seems to be very promising for solving this problem. The Notch signaling pathway is found in many cell types (Artavanis-Tsakonas et al., 1999; Fortini, 2009). Recently, the activation of Notch signaling pathway was found to be participating in the pathogenesis of many types of cancer such as T-cell leukemia, lymphoma, medulloblastoma and colorectal, pancreatic, mammary, ovarian, lung, gastric, cervical and breast carcinoma (Moserle et al., 2010; Wu et al., 2010; Yin et al., 2010; Meurette and Mehlen, 2018; kontomonalis et al., 2018). Accordingly, applying Notch inhibitors may be promising in the treatment of such diseases. Besides, small molecule inhibitors for gamma-secretase activity have been actively investigated, over the past decades, for their potential to block the generation of Aβ-peptide that is associated with Alzheimer’s disease (Bateman et al., 2009). Because gamma-secretase inhibitors are also able to effectively inhibit Notch receptor signaling, several forms of gamma-secretase inhibitors, including N-[N-(3,5-difluorophenacetyl)-l-alanyl]-Sphenylglycine t-butyl ester (DAPT), compound E, and IL-X (cbz-IL-CHO) MRK-003 and DBZ, have been tested for the treatment of tumor and cardiovascular diseases (Aoyama et al., 2009; Kang et al., 2009; Acharya et al., 2011).

The notch pathway was shown to be critically involved in some renal disorders (Huang et al., 2011). Nevertheless, the possible role of the Notch pathway in cisplatin nephrotoxicity has not been studied before, which was found to be an interesting point to be explored. In this study, the mRNA levels of Notch-1 receptor and Hes-1 were markedly elevated in the cisplatin-injected group. Being a gamma-secretase inhibitor, the DBZ pre-treatment significantly attenuated the increase in the translational levels of the assessed Notch pathway molecules. Also, the TNF-α stimulated NF-κB signaling, in collaboration with the basal Notch signals, affects the expression of the Notch targets. Mechanistically, TNF-α induces the phosphorylation of histone H3 at the Hes1 promoter. Also, a crosstalk between TNF-α/NF-κB and Notch was found to sustain the intrinsic inflammatory profile of the transformed cells (Maniati et al., 2011). In addition, Hes-1 was shown to induce NF-κB gene transcription, which links the Notch pathways signaling with inflammation (Cao et al., 2010). Furthermore, for studying the modulatory effects of DBZ on cisplatin cytotoxic activity, DBZ was used at a concentration that inhibits only 5% of the cancer cells which was calculated to be 10 μM. From the concentration-response survival curves, it was found that the pre-treatment with DBZ for 24 h showed no significant changes in cisplatin cytotoxicity on PC3 and Hela cancer cells.

In conclusion, all these results suggest that DBZ exerts a promising nephroprotective effects against cisplatin-induced acute renal injury in rats, where it greatly improved the nephrotoxicity markers. These protective effects of DBZ were achieved by reducing oxidative stress, inflammation and apoptosis. Interestingly, the Notch signaling pathway was shown to get activated upon cisplatin administration, a result which was reversed by DBZ. Additionally, cisplatin cytotoxic activity was preserved by the pre-treatment with subtoxic concentration of DBZ, *in vitro*, in PC3 and Hela human cancer cell lines.

Taken together, these findings indicate that the use of DBZ may provide nephroprotection without affecting cisplatin cytotoxic activity. The pre-treatment of cisplatin-injected rats with DBZ markedly reduced the oxidative stress, inflammation as well as apoptotic markers. Moreover, these findings reflect, for the first time, the possible role that the Notch pathway may be playing in the pathogenesis of cisplatin nephrotoxicity. This paves the way for further investigations of the Notch pathway in cisplatin-induced nephrotoxicity in more mechanistic details. Also, these findings make it promising to try other Notch inhibitors for their possible nephroprotective properties in future studies.

## Data Availability Statement

The original contributions presented in the study are included in the article/Supplementary Material, further inquiries can be directed to the corresponding author.

## Ethics Statement

The animal study was reviewed and approved by The Ethical Committee of Faculty of Pharmacy, Ain shams University.

## Author Contributions

RE-N designed the experiments. RAE-R performed the experiments. RE-N and AG supervised the experimental part RAE-R, RE-N, and AG analyzed the data. RAE-R contributed the reagents/materials/analysis tools. RAE-R, RE-N, and AG wrote the manuscript. MT and SH revised the manuscript.

## Conflict of Interest

The authors declare that the research was conducted in the absence of any commercial or financial relationships that could be construed as a potential conflict of interest.
